# Supplementary material for: Assessment of Popcorn’s Bioactive Status in Response to Popping
Source: Molecules. 2024 Feb 9;29(4):807. doi: 10.3390/molecules29040807 (PMC10891987; doi:10.3390/molecules29040807)
Supplement: Supplementary file 1 [file molecules-29-00807-s001.zip › molecules-2819060-supplementary.pdf]

Figure S1: The obtained eigenvalues and cross-validation results for the analysed data.

Eigenvalues and Cross-validation Results for data - DataSet Editor

File Edit Transform View FigBrowser

Info Data Plot Row Labels Column Labels

Label Set 1 >	1: Eigenvalues	2: Variance Captured (%)	3: Cumulative Variance Captured (%)	4: ln(eigenvalues)	5: log(eigenvalues)	6: Eigenvalue Ratio	7: RMSECV	8: RMSEC
1: 1	3.5395	39.328	39.328	1.264	0.54894	2.2802	2.9328	0.76252
2: 2	1.5523	17.2476	56.5756	0.43973	0.19097	1.2484	3.3265	0.6451
3: 3	1.2434	13.8156	70.3912	0.21785	0.094612	1.1475	3.5099	0.53268
4: 4	1.0835	12.0394	82.4306	0.080242	0.034849	1.3738	4.1423	0.41033
5: 5	0.78874	8.7637	91.1943	-0.23732	-0.10307	2.3012	4.3078	0.2905
6: 6	0.34275	3.8083	95.0026	-1.0708	-0.46503	1.2122	6.531	0.21884
7: 7	0.28276	3.1417	98.1444	-1.2632	-0.54859	2.5992	9.5911	0.13335
8: 8	0.10879	1.2087	99.3531	-2.2184	-0.96342	1.8685	21.2863	0.078737
9: 9	0.058221	0.6469	100	-2.8435	-1.2349	NaN	11.3493	1.5232e-015

^ Principal Component Number
